# Hypofibrinogenemia Induced by Eravacycline: A Rare but Serious Adverse Effect of a Novel Antimicrobial Agent

**DOI:** 10.7759/cureus.107424

**Published:** 2026-04-20

**Authors:** Pradeep Khanal, Suhail Sapkota, Ioannis Karageorgiou, Tareq Al Baghdadi

**Affiliations:** 1 Hematology and Oncology, Henry Ford Health, Detroit, USA; 2 Internal Medicine, University of Michigan Health-Sparrow, Lansing, USA; 3 Internal Medicine, Corewell Health William Beaumont University Hospital, Royal Oak, USA; 4 Hematology and Oncology, St Joseph Mercy Hospital, Ypsilanti, USA

**Keywords:** acquired hypofibrinogenemia, drug-induced coagulopathy, eravacycline, fibrinogen monitoring, tetracycline

## Abstract

Eravacycline is a broad-spectrum third-generation tetracycline, notably effective against multidrug-resistant organisms. Emerging evidence suggests an association with hypofibrinogenemia, though clinical recognition remains limited. We report a case of an 84-year-old male patient who presented with hypofibrinogenemia (fibrinogen <35 mg/dl) while being on eravacycline for *Stenotrophomonas maltophila* diabetic foot infection in the right toe. Alternative etiologies, including disseminated intravascular coagulation (DIC) and hepatic dysfunction, were excluded. Immediate discontinuation of the antibiotic and cryoprecipitate infusions resulted in improvement and stabilization of fibrinogen levels. This case highlights the clinically meaningful and likely underrecognized adverse effect and supports monitoring fibrinogen and other coagulation parameters during treatment.

## Introduction

Hypofibrinogenemia is defined as a quantitative fibrinogen disorder characterized by reduced plasma fibrinogen levels (typically <200 mg/dL), which can be either congenital or acquired. Eravacycline is a synthetic third-generation tetracycline approved by the Food and Drug Administration in 2018 and has also been approved by the European Medicines Agency. It has broad-spectrum antimicrobial activity against Gram-positive, Gram-negative, and anaerobic bacteria, including several clinically significant multidrug-resistant (MDR) organisms [1,2]. Its mechanism of action involves inhibiting bacterial protein synthesis by binding to the 30S ribosomal subunit, preventing amino acid incorporation into the peptide chain [2]. Gastrointestinal adverse effects, including nausea and vomiting, as well as infusion-related reactions, were the most commonly reported in phase 2/3 clinical trials [3,4]. While tigecycline-associated hypofibrinogenemia is well documented, emerging reports suggest a similar safety signal with eravacycline, though severe symptomatic cases remain limited in the literature [5-10]. Current evidence is limited to case reports and small retrospective studies, with reported incidence of hypofibrinogenemia in up to 40-50% of patients receiving eravacycline, although most cases are mild. This case report describes an older male patient who developed severe hypofibrinogenemia attributed to eravacycline.

## Case presentation

**Figure 1:**
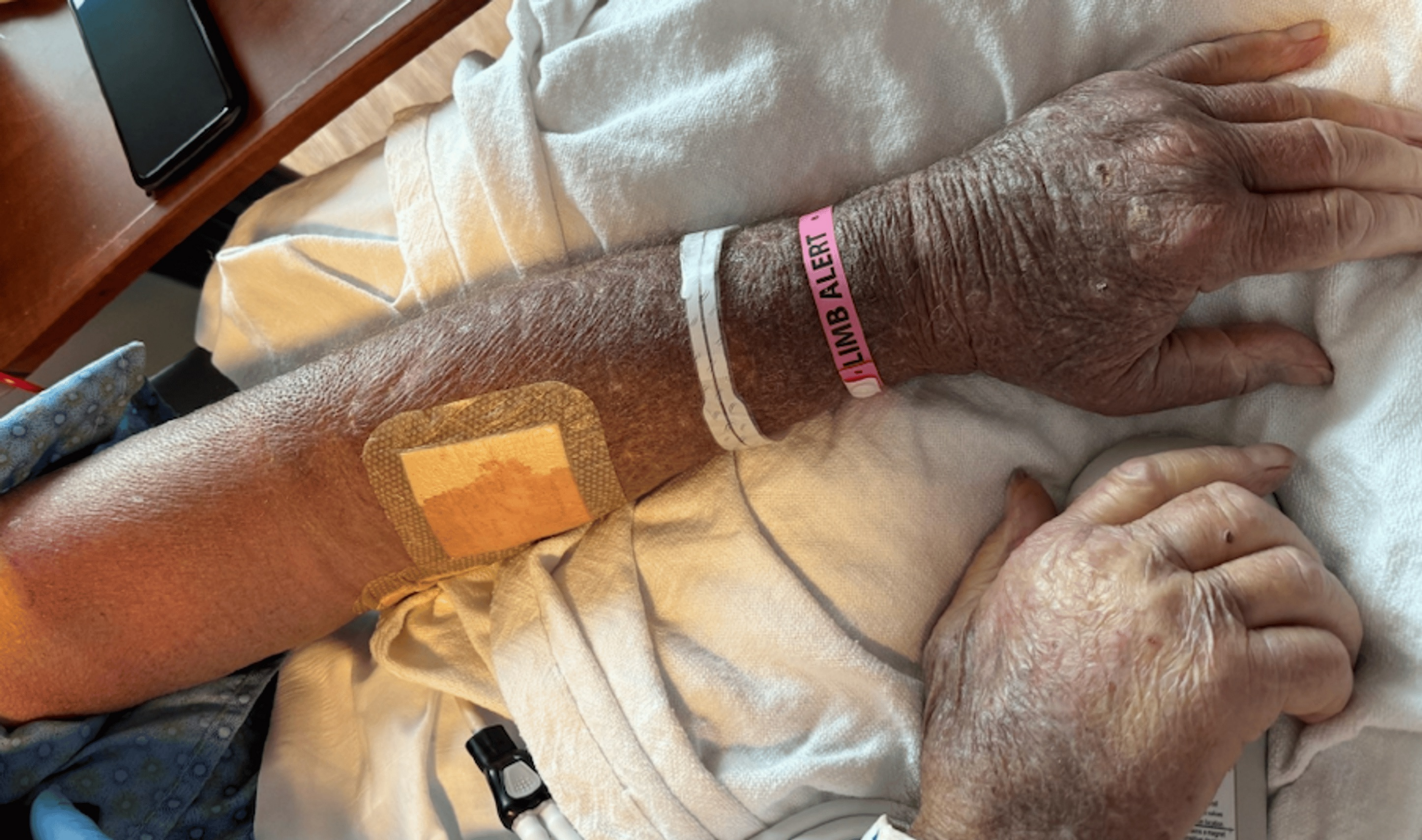
Ecchymosis and swelling of the left upper extremity Clinical photograph demonstrating diffuse ecchymosis and swelling of the left upper extremity in the setting of severe hypofibrinogenemia.

An 84-year-old male patient presented to the emergency department with a chief complaint of left upper extremity swelling and bruising, ongoing for three weeks. His past medical history included type 2 diabetes mellitus, chronic kidney disease stage IIIa, coronary artery disease, and chronic heart failure with a reduced ejection fraction. He was receiving a four-week antibiotic course of IV eravacycline 80 mg (1mg/kg) twice daily, for the treatment of *Stenotrophomonas maltophila* cultured from a diabetic foot ulcer on his right toe. The patient developed symptoms approximately seven days after initiation of eravacycline therapy. On examination, he was alert, oriented, and hemodynamically stable. His left upper extremity appeared swollen and ecchymotic, as shown in Figure 1. 

Laboratory evaluation revealed severe hypofibrinogenemia (<35 mg/dL), and prothrombin time (PT), international normalized ratio (INR), and activated partial thromboplastin time (aPTT) were unmeasurable. Additional findings included anemia and thrombocytopenia. Hemolysis workup was performed to evaluate for microangiopathic processes and revealed low haptoglobin (25 mg/dL) and mildly elevated lactate dehydrogenase (406 units/L); however, no schistocytes were identified on peripheral smear, and the direct antiglobulin test was negative. Liver function tests showed a mild elevation of aspartate aminotransferase (AST) at 45 U/L and total bilirubin at 1.2 mg/dL. Factor VIII activity, von Willebrand factor antigen, and von Willebrand factor activity ristocetin test were within normal limits (Table 1).

**Table 1:** Laboratory findings on presentation WBC: white blood cell count; aPTT: activated partial thromboplastin time; INR: international normalized ratio; Unmeasurable values indicate inability to detect clot formation due to severe hypofibrinogenemia.

Test	Result	Reference range
Fibrinogen	<35 mg/dL	195 - 495 mg/dL
Hemoglobin	8.6 g/dL	13.5 - 17.5 g/dL
WBC	8.9 × 10³/μL	4 - 10 × 10³/μL
Platelet count	76 k/mcL	140 - 450 k/mcL
D-dimer	581 ng/mL	≤230 ng/mL
Haptoglobin	25 mg/dL	36 - 215 mg/dL
Lactate dehydrogenase	406 U/L	142 - 271 U/L
Aspartate aminotransferase	45 U/L	13 - 39 U/L
Total bilirubin	1.2 mg/dL	0.3 - 1 mg/dL
aPTT	Unmeasurable	21 - 31 s
Prothrombin time	Unmeasurable	9.5 - 12.1 s
INR	Unmeasurable	0.8 - 1.2

Left upper extremity venous duplex revealed acute superficial thrombophlebitis of the cephalic vein. CT angiography of the chest, abdomen, and pelvis with and without contrast were unremarkable for an acute infectious process. Representative imaging was not available for inclusion. Infectious workup, including COVID-19, Influenza A and B, respiratory syncytial virus testing, and blood cultures, was negative. 

Low fibrinogen, mildly elevated D-dimer, thrombocytopenia, and anemia raised concern for disseminated intravascular coagulation (DIC). An International Society on Thrombosis and Hemostasis (ISTH) DIC score was not consistent with overt DIC based on available laboratory parameters and clinical context. Acute liver failure was also considered due to low fibrinogen, thrombocytopenia, and low haptoglobin, but this was unlikely due to only mild elevation in both aspartate aminotransferase (AST) and total bilirubin. The patient did not exhibit clinically significant bleeding despite severe hypofibrinogenemia. The temporal association with eravacycline exposure, absence of alternative etiologies, and rapid improvement following drug discontinuation supported a probable drug-induced etiology. 

Eravacycline was promptly discontinued. Over the first three days of hospitalization, the patient received a total of six units of cryoprecipitate to maintain a fibrinogen level ≥125 mg/dL. The patient’s fibrinogen had normalized by day eight of hospitalization and remained stable, with no further indication for transfusion. His platelet count improved to baseline, and hemoglobin remained stable above 7 g/dL. The diabetic foot infection of the right toe had resolved by the time of this admission, and his superficial thrombophlebitis of the left upper extremity was managed conservatively with elevation and cold compresses. The patient remained hemodynamically stable and was transferred to a skilled nursing facility for rehabilitation after a nine-day hospital stay. 

## Discussion

Hypofibrinogenemia is a rare and potentially serious side effect of eravacycline that warrants careful consideration. Several case reports have documented hypofibrinogenemia with tigecycline, a related tetracycline antibiotic, with similar coagulopathies reported in both agents. This suggests that this side effect might be a class effect of synthetic tetracyclines [8,9]. Tigecycline-induced hypofibrinogenemia typically manifests as a dose-dependent reduction in fibrinogen levels, often accompanied by prolonged coagulation times, including PT and aPTT [8,9]. Previous studies have shown that tigecycline can lead to hypofibrinogenemia within a few days of treatment initiation, with fibrinogen levels often declining to dangerously low levels [7,9]. 

A recent retrospective study found that nearly half of patients receiving eravacycline developed hypofibrinogenemia (<200 mg/dL), although none had major bleeding [10]. In contrast, our case demonstrated a severe and symptomatic presentation, emphasizing the potential clinical significance of this association. In this case, advanced age and chronic kidney disease may have contributed to increased susceptibility to this adverse effect.

The mechanism by which eravacycline causes hypofibrinogenemia remains unclear. One hypothesis suggests that these drugs interfere with fibrinogen synthesis by affecting liver function [9]. Another possible mechanism is direct inhibition of the coagulation cascade, which may be exacerbated by underlying conditions such as liver or renal impairment, that alter drug clearance and increase the risk of coagulopathy [8,9]. Its structural similarity to tigecycline also raises the possibility of a class-related effect among fluorocyclines [9].

Although the FDA label notes prolonged aPTT as a rare adverse effect (<1%), it does not specifically address hypofibrinogenemia [11]. Stopping eravacycline promptly and giving cryoprecipitate led to the recovery of fibrinogen levels and correction of the coagulopathy, highlighting the value of early recognition and timely supportive management [7]. We suggest baseline fibrinogen assessment, followed by repeat testing within three to five days and weekly thereafter for prolonged therapy, particularly in patients receiving longer courses.

As eravacycline and other tetracyclines are used more often for multidrug-resistant infections, there is a clear need to better understand the mechanisms behind antibiotic-associated hypofibrinogenemia and to define practical strategies that reduce this risk without compromising treatment effectiveness. Further research should aim to identify patients at higher risk for this complication and to clarify when and how coagulation parameters should be monitored during therapy [7,8].

## Conclusions

This case emphasizes the rare but serious risk of hypofibrinogenemia associated with eravacycline therapy. Healthcare providers must be aware of this potential complication, especially in higher-risk individuals, including those with renal and hepatic dysfunction and advanced age, and ensure close monitoring of coagulation parameters in patients receiving this agent. Early recognition and management of hypofibrinogenemia, including discontinuation of the offending drug and supportive care, are crucial for preventing adverse complications and ensuring a favourable outcome.
